# Unphazed and Phogo: complete genome sequences of two EK1 cluster *Podoviridae Microbacterium* phages containing putative purple acid phosphatase genes

**DOI:** 10.1128/mra.00423-26

**Published:** 2026-08-03

**Authors:** Brett M. Condon, Jacob D. Ariel, Saundra L. Godshall, Drithi Patel, Drexel University SEA-PHAGES annotators 2020, Minahil Chaudhry, Matthew T. McDonald, Susan M. R. Gurney

**Affiliations:** 1 Department of Biomedical Sciences, Rocky Vista University–Montana College of Osteopathic Medicine, Billings, Montana, USA; 2 Lewis Katz School of Medicine, Temple University 12314, Philadelphia, Pennsylvania, USA; 3 Bristol Myers Squibb, Princeton, New Jersey, USA; 4 Department of Radiation Oncology, Thomas Jefferson University 199720, Philadelphia, Pennsylvania, USA; 5 Department of Biology, Drexel University 219255, Philadelphia, Pennsylvania, USA; 6 School of Biology, University of St Andrews, Fife, United Kingdom; Portland State University, Portland, Oregon, USA

**Keywords:** bacteriophage, genomes, EK1 cluster, *Microbacterium foliorum*

## Abstract

We report two EK1 cluster bacteriophages isolated from soil samples using the host *Microbacterium foliorum*. EM imaging showed that both exhibit the podovirus morphology. Genome sequencing and annotation showed similarity with many EK1 phage genomes. Our phages contain a putative PAP gene, also expressed in plants.

## ANNOUNCEMENT

Members of *Microbacterium foliorum* (*M. foliorum*; NRRL B-23224) are found in diverse environments, including on the surface of plants (1, 2). Plants evolved purple acid phosphatases (PAPs) to enhance phosphorus acquisition from soil that would otherwise limit plant growth (3, 4). We report the isolation of two *M. foliorum* bacteriophages that contain a putative PAP gene.

**Fig 1:**
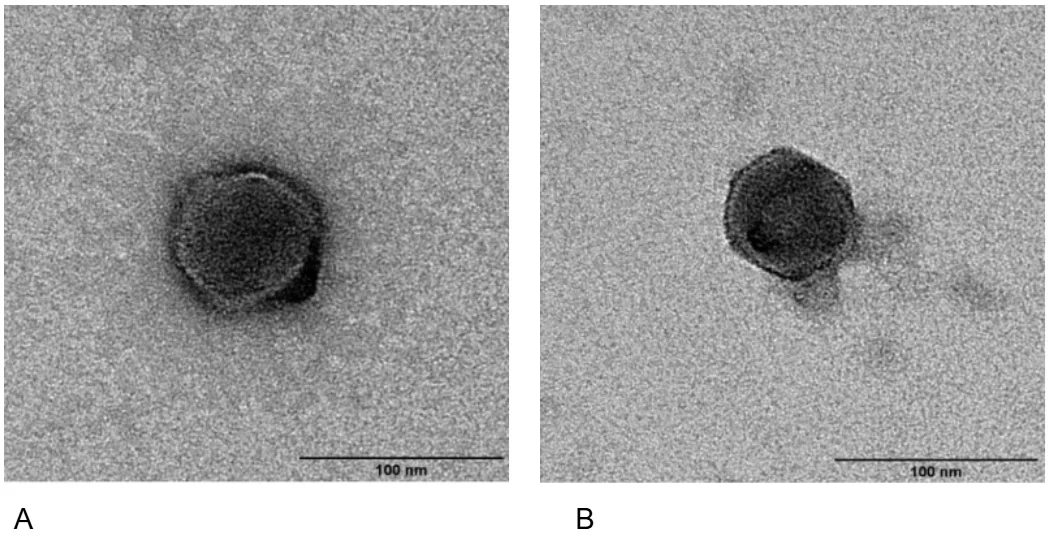
Transmission electron microscopy images of bacteriophages: (**A**) Unphazed and (**B**) Phogo. The samples were stained with 1% uranyl acetate and imaged using a JEOL 2100F field-emission transmission electron microscope.

Our phages were isolated from soil in Philadelphia, using *M. foliorum*. Both were imaged by negative stain transmission electron microscopy and exhibit short non-contractile tails (Fig. 1) consistent with the podovirus morphology. Phages with podovirus morphology are found in the EK cluster (5, 6), 1 of 21 microbacterium phage clusters organized by sequence similarity.

Following the “Direct Isolation” protocol (7), soil samples were collected (Table 1), mixed with PYCa media, and shaken for 2 h at 30°C. The medium was filtered (with a 0.22 µm syringe filter), and the filtrate plated in PYCa top agar with *M. foliorum* and incubated at 30°C for plaque formation. Plaques were purified through three rounds of plating, after which lysates were prepared from webbed plates. DNA was extracted from lysate using the Promega Wizard DNA cleanup kit (7). Sequencing libraries were prepared from genomic DNA with an NEB Ultra II FS Kit. Genomes were sequenced with the Illumina MiSeq platform (V3 reagents). Raw reads were assembled with Newbler 2.9 (8), each yielding a single phage contig, which was checked for completeness, accuracy, and phage genomic termini with Consed 29 (9, 10) (see Table 1). Sequenced genomes were annotated with the following tools: DNA Master 5.23.3 (11), Glimmer 3.02 (12), GeneMark 2.5p (13), Starterator (https://phages.wustl.edu/starterator/), Phamerator (using Actino_draft database v578) (14), PhagesDB BLAST (https://phagesdb.org/blast/), NCBI BLASTP (using the Actinobacteriophage and NCBI non-redundant database) (15), the NCBI CDD (16), HHPRED (using the PDB_mmCIF70, Pfam- v.36, NCBI Conserved Domains databases) (17), Aragorn 1.2.38 (18), tRNAscanSE 2.0 (19), PECAAN (https://discover.kbrinsgd.org), and manual inspection. Default settings were used on all software. Both phages were assigned to the cluster EK1 based on gene content similarity of at least 35% to phages in the Actinobacteriophage database, phagesdb (https://phagesdb.org) (20, 21).

**TABLE 1:** Soil collection and sequencing information for EK1 phages

Phage name	Location	GPS	Depth of soil	Date of collection	Subcluster	GenBank accession number	SRA accession number	Length (bp)	G + C (%)	Protein coding genes (#)	Genome sequencing coverage	Number of reads
Unphazed	Philadelphia, PA, USA	39.9529°N, 75.1912°W	3–8 cm	9/9/2019	EK1	MT310887	SRX31360684	53,919	60.2	54	286	193,481
Phogo	Philadelphia, PA, USA	39.9608°N, 75.1879°W	3–8 cm	9/9/2019	EK1	MT310888	SRX31360680	53,959	60	54	26	212,574

Phogo and Unphased are both predicted to encode a putative purple acid phosphatase gene (gene number 47, phamily number 270,990). Of the 25 phages in this subcluster to date, 15 phages, including Phogo and Unphazed, encode this gene “phamily” (22). Of the 10 phages that lacked this phamily, 5 encoded a “phosphoesterase,” and 5 had no corresponding gene at this location (14). Other than Rowlf (23) (EG cluster bacteriophage) and A3Wally (GD cluster bacteriophage), only EK cluster bacteriophages are known to encode this PAP family. While PAP has a clear functional role in plant growth (3, 4), its function in bacteria is limited, and in bacteriophages, it is unknown (24).

While a growing number of scientists are engaged in sequencing and annotating bacteriophage genomes, more functional studies on bacteriophage genes are needed.

## Data Availability

Accession numbers for these EK1 cluster genome sequences are provided in Table 1, under BioProject number PRJNA488469.
